# Syncope in AQP4-IgG-positive neuromyelitis optica spectrum disorder: a case series and literature review

**DOI:** 10.3389/fnins.2026.1722763

**Published:** 2026-03-11

**Authors:** Chenyang Jin, Yuyuan Yang, Xiaoqian Song, Yuewen Sun, Yilong Peng, Liyan Wang, Xueping Zheng

**Affiliations:** 1 Department of Geriatric Medicine, The Affiliated Hospital of Qingdao University, Qingdao, China; 2 Qingdao Medical College, Qingdao University, Qingdao, China; 3 Third People’s Hospital of Liaocheng, Liaocheng, China

**Keywords:** AQP4, area postrema syndrome, autonomic dysfunction, medulla oblongata, NMOSD, syncope

## Abstract

**Objective:**

To report two cases of aquaporin-4 immunoglobulin G (AQP4-IgG)-positive neuromyelitis optica spectrum disorder (NMOSD) with syncope and to review previously documented cases, aiming to enhance recognition of this neurological manifestation and guide timely intervention.

**Methods:**

We reported two NMOSD cases presenting with syncope. A literature search was conducted in PubMed using Medical Subject Headings (MeSH) and relevant keywords related to NMOSD, area postrema syndrome, and syncope.

**Results:**

The two patients, a 40-year-old man and a 41-year-old woman, presented with area postrema syndrome (APS) and recurrent syncope. The syncope attacks were transient, characterized by sudden loss of consciousness and spontaneous recovery. Electrocardiograms on admission were unremarkable in both cases. MRI showed dorsal medullary oblongata lesions. Both were treated with high-dose intravenous methylprednisolone followed by maintenance inebilizumab, leading to complete clinical remission, seroconversion to AQP4-IgG negativity, and an Expanded Disability Status Scale (EDSS) score of 0 at last follow-up. Besides, a total of 16 cases were identified and reviewed in this study, including our cases. The cohort showed equal sex distribution (male-to-female ratio 1:1) and a mean age of 53.94 years. All patients presented with APS preceding syncope, and neuroimaging revealed medulla oblongata lesions. Electrocardiographic abnormalities were documented in eight patients, all presenting with sinus arrest. Considering the possible mechanisms of syncope in these patients, eight patients had sinus arrest, two had orthostatic hypotension, and two experienced cardiorespiratory arrest. Among the eight patients with sinus arrest, six underwent cardiac interventions (75%): TPM implantation in four (one also undergoing cardioneuroablation) and PPM implantation in two. Two patients (25%) achieved remission with IVMP alone without cardiac intervention. All 16 patients remained free of syncope during follow-up.

**Conclusion:**

Syncope may be an underrecognized manifestation of AQP4-IgG-positive NMOSD. Patients presenting with APS should be screened for syncope attacks, which may require emergency pacemaker implantation.

## 1 Introduction

Neuromyelitis optica spectrum disorder (NMOSD) is an immune-mediated disease associated with anti-aquaporin-4 immunoglobulin G (AQP4-IgG) (Wingerchuk et al., 2015), with a 3 to 5-fold higher prevalence in East Asian populations compared to Caucasians (Hor et al., 2020). NMOSD manifests with a broad spectrum of clinical symptoms, classically including optic neuritis, acute myelitis, and area postrema syndrome (APS). APS is clinically defined as unexplained episodes of hiccups, nausea, or vomiting persisting for more than 48 h (Huda et al., 2019). Syncope is typically regarded as a manifestation of primary cardiac disorders (Chen et al., 2025). However, recent studies have increasingly recognized syncope as an associated symptom in patients with NMOSD (Yin et al., 2025).

Owing to the limited number of reported cases, syncope remains underrecognized in the clinical management of NMOSD. This oversight may contribute to diagnostic uncertainty and delayed treatment. To better characterize the clinical manifestations, potential pathogenic mechanisms, and therapeutic strategies for patients with these coexisting conditions, we reported two cases of NMOSD with syncope and reviewed a total of 16 such cases from the literature.

## 2 Case reports

### 2.1 Case 1

**Figure 1:**
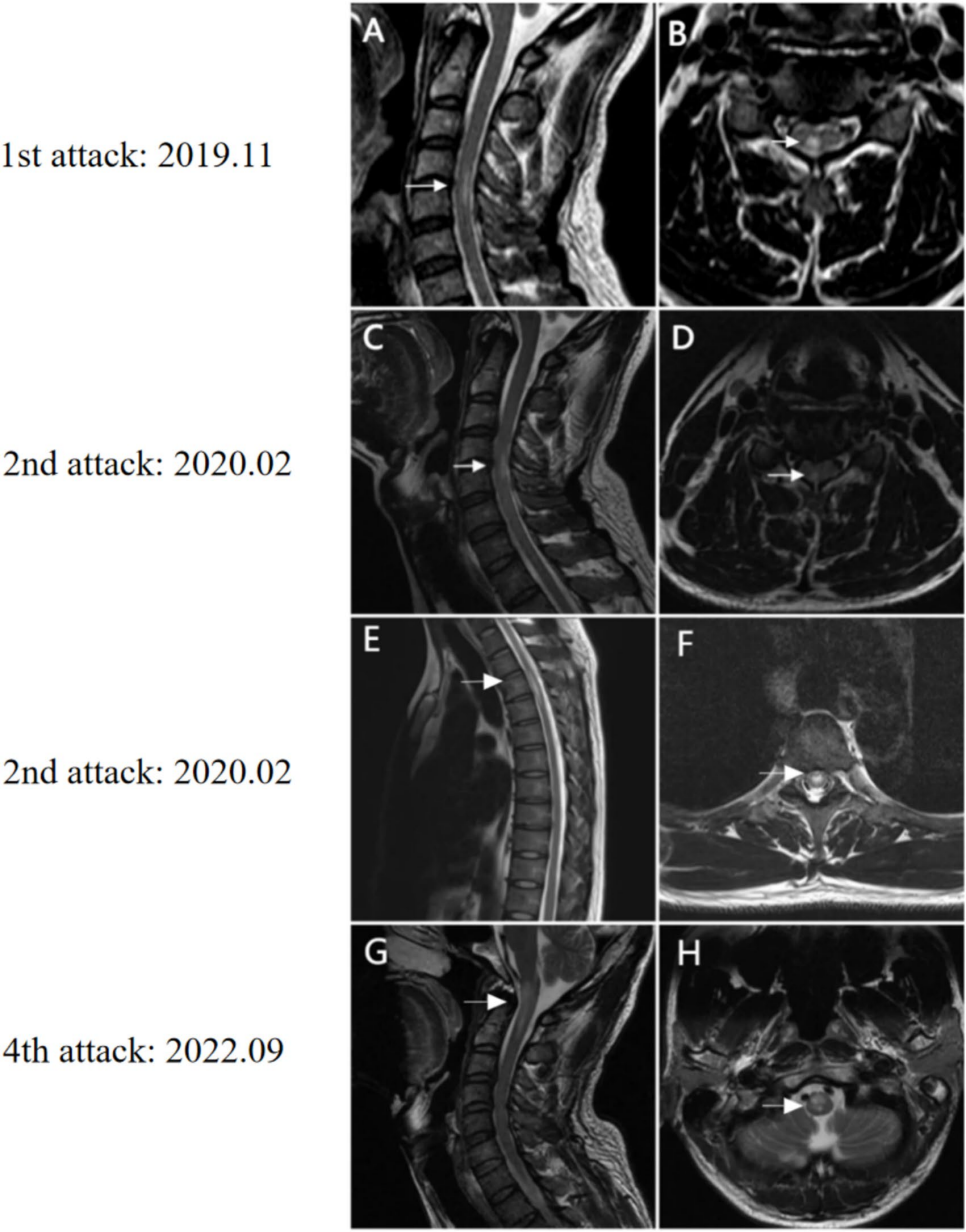
MRI findings of Case 1. **(A)** Sagittal T2-weighted cervical spinal cord MRI at first presentation shows a short-segment hyperintense lesion at the C4–C5 level (arrow). **(B)** Axial image demonstrating posterior spinal cord involvement (arrow). During the second attack, sagittal T2-weighted image **(C)** reveals C4–C5 hyperintensity (arrow), while axial imaging **(D)** shows posterior cord parenchymal involvement (arrow). Sagittal **(E)** and axial **(F)** T2-weighted images of the thoracic spinal cord demonstrate a new lesion at T4–T6 (arrows). Sagittal T2-weighted image **(G)** demonstrates a hyperintense lesion extending from the medulla oblongata to C2 (arrow), with **(H)** axial view confirming medullary involvement (arrow).

A 40-year-old man initially presented with a two-day history of right-sided limb numbness in November 2019. The patient exhibited no mobility limitations, and Expanded Disability Status Scale (EDSS) score was 1. Brain and cervical MRI demonstrated abnormal hyperintense signal lesions in the posterior column of the spinal cord at the C4–C5 level (Figure 1). He was diagnosed with “acute myelitis.” Intravenous methylprednisolone (dose not described) was administered at a local hospital, which led to complete resolution of symptoms. Following discharge, no maintenance medication was continued to this patient.

In February 2020, the patient was admitted in our hospital with a ten-day history of bilateral ocular pain and a two-day history of blurred vision in the right eye. One day prior to admission, he developed new-onset numbness in the left lower limb and chest pain. Neurological examination revealed a corrected visual acuity of 0.1 in the right eye and 1.0 in the left eye, with a right-sided visual field defect. Muscle strength and tone were normal. Sensory abnormalities included hypoalgesia below the right knee and hyperalgesia in T1–T7. EDSS score was 3. Orbital CT showed right optic nerve thickening. Cervical MRI showed a chronic lesion at the C4–C5 level, whereas a new lesion was detected at the T3–T4 thoracic level on spinal MRI (Figure 1). Brain MRI showed no significant findings. Serum tests for extractable nuclear antigen (ENA) antibodies, anti-neutrophil cytoplasmic antibodies, and anti-nuclear antibody tests were all negative. Cerebrospinal fluid (CSF) analysis revealed mild increase of white blood cell (48/mm^3^, normal range 0–6/mm^3^), and normal protein concentration. Serum AQP4-IgG testing was positive with a titer of 1:1,000 (cell-based assay), confirming the diagnosis of NMOSD according to the 2015 International Panel diagnostic criteria (Wingerchuk et al., 2015). Then, he was administered intravenous methylprednisolone (500 mg/day) for 5 days, with a gradual reduction to oral dose. His symptoms improved substantially, and EDSS score decreased to 2. After discharge, the patient was maintained on oral prednisone therapy for approximately 6 months.

In May 2022, the patient presented with dizziness accompanied by intractable nausea, vomiting, hiccups, and lower limb weakness. He reported several transient episodes of loss of consciousness. On admission, the heart rate was 76 bpm, blood pressure was 119/88 mmHg, and the electrocardiogram (ECG) was unremarkable. His EDSS score was 3. Brain MRI was normal. However, cervical spine MRI was not performed. No systematic assessment of autonomic function, such as orthostatic blood pressure measurement or head-up tilt testing, was conducted. The recurrent syncope typically occurred after vomiting, and lasted up to three minutes, without urinary or fecal incontinence. Although a comprehensive syncope evaluation, including 24-h Holter monitoring, was not performed during this admission, prior Holter recordings had been unremarkable.

**Figure 2:**
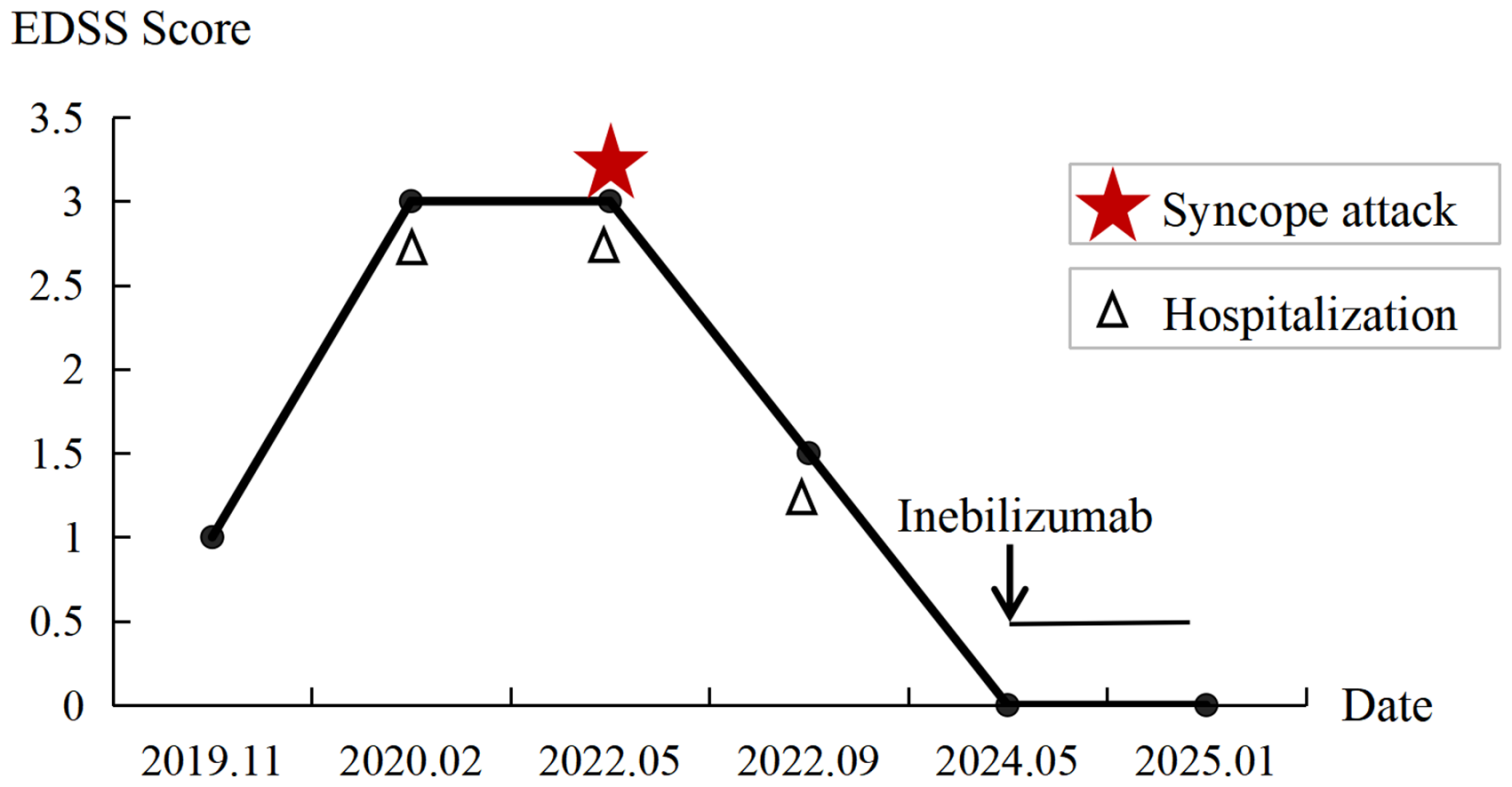
Expanded Disability Status Scale (EDSS) score and clinical course of Case 1. The EDSS was 1.0 at the first episode. Three relapses occurred between February 2020 and September 2022, with syncope presenting during the relapse in May 2022 (red star). The patient received inebilizumab in May 2024, and the EDSS score was 0 at last follow-up.

In September 2022, the patient experienced a recurrence of symptoms, including right-sided headache, nausea, and vomiting without syncope. His EDSS score was 1.5. Cervical MRI demonstrated abnormal signal intensity extending from the medulla oblongata to the C2 vertebral level (Figure 1). He received intravenous methylprednisolone pulse therapy (IVMP, 1,000 mg/day for 5 days), followed by a gradual taper, with marked clinical improvement. The patient was maintained on oral prednisone in combination with tacrolimus, which achieved disease stability but was complicated by treatment-related adverse effects, including elevated blood glucose levels. Therefore, in May 2024, inebilizumab therapy was initiated (300 mg intravenously on days 0 and 15, followed by maintenance dosing every 6 months). No relapses occurred and AQP4-IgG antibody has been seronegative to date. EDSS score was 0 (Figure 2).

### 2.2 Case 2

**Figure 3:**
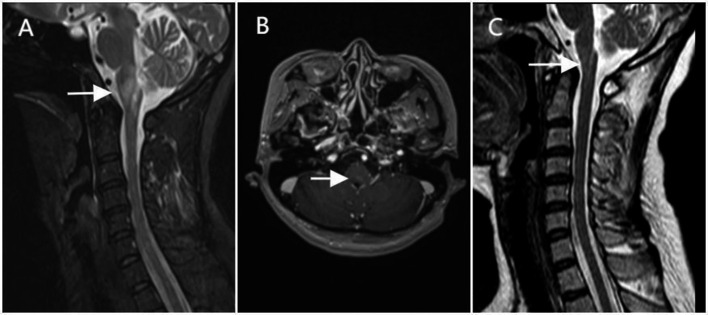
MRI findings of Case 2. A 41-year-old woman with area postrema syndrome (initial presentation in July 2023). **(A)** Sagittal T2-weighted image shows a hyperintense lesion (arrow) in the area postrema of the medulla oblongata. **(B)** Axial contrast-enhanced T1-weighted image demonstrates mild enhancement of the lesion (arrow) in the medulla oblongata. **(C)** Follow-up sagittal T2-weighted cervical spine image (September 2024) reveals reduction in the size of the lesion (arrow) compared with the initial presentation.

A 41-year-old woman was admitted in July 2023 with one-month history of nausea, vomiting, persistent hiccups and dizziness. In the eight days preceding hospital admission, she experienced two brief episodes of loss of consciousness, each lasting a few seconds, without urinary or fecal incontinence or limb convulsions. On admission, her vital signs were stable with a heart rate of 80 bpm, blood pressure of 137/75 mmHg, and the ECG was normal. A detailed assessment of autonomic function and 24-h Holter monitoring was not performed. Physical examination revealed horizontal nystagmus, bilateral lower limb strength grade 4 and hypoalgesia below the T12 level. Her EDSS score was 3.5. Serum autoantibody testing revealed strong positivity for anti-Sjögren’s syndrome-related antigen A (anti-SSA, +++), anti-Ro-52 (+++), and positivity for anti-SSB (+) antibody. CSF examination showed markedly elevated white blood cells (132/mm^3^), mildly elevated protein (668.0 mg/L, reference 120–600 mg/L). Serum AQP4-IgG was positive with a titer of 1:32 (cell-based assay). Cervical MRI showed abnormal signal in the area postrema (AP) (Figure 3), whereas thoracic spinal was unremarkable. Based on the positive AQP4-IgG findings and characteristic clinical features, the patient was diagnosed with NMOSD. However, the patient was not diagnosed with Sjögren’s syndrome because she lacked sicca symptoms (xerophthalmia or xerostomia).

**Figure 4:**
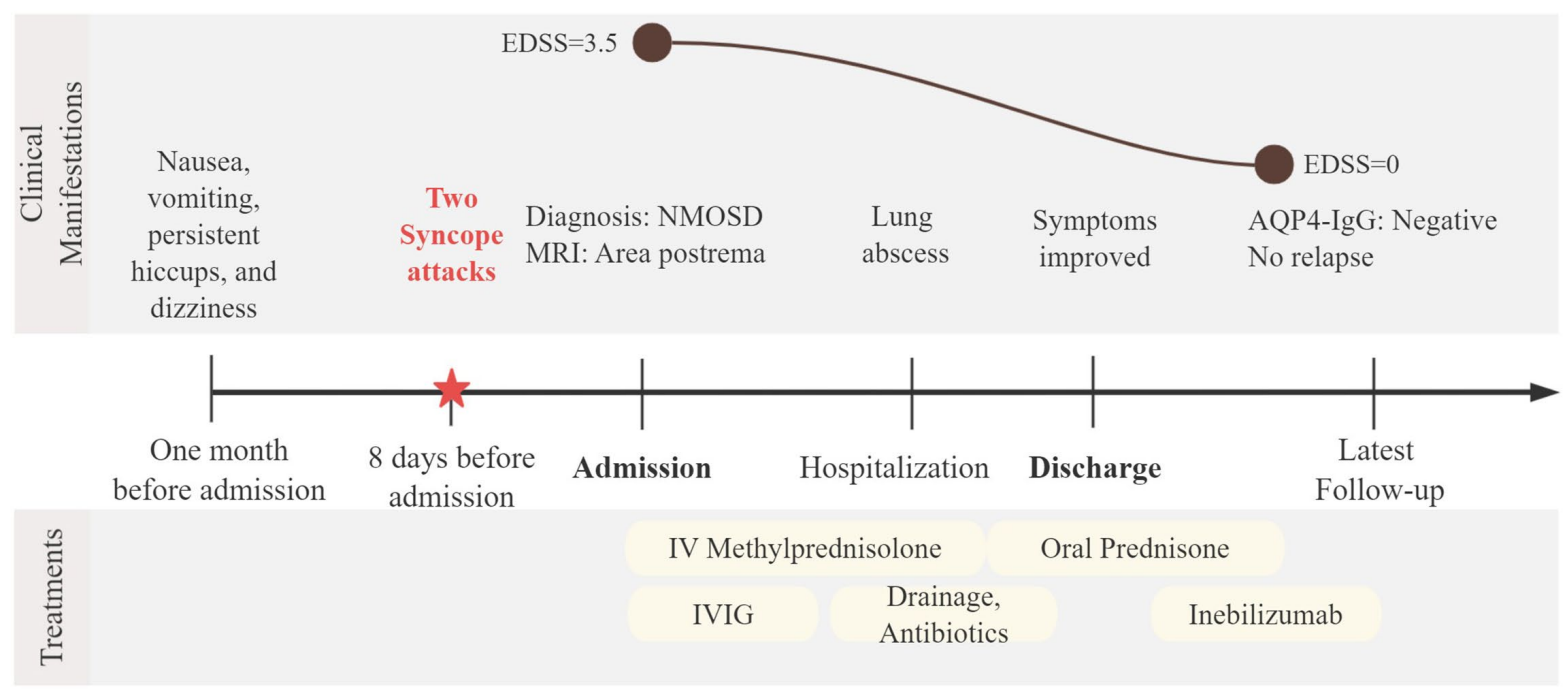
Timeline of clinical course in Case 2. A 41-year-old woman developed area postrema syndrome with two brief syncopal episodes before admission and had an Expanded Disability Status Scale (EDSS) score of 3.5, with an area postrema lesion on cervical MRI. She received intravenous methylprednisolone and intravenous immunoglobulin (IVIG), complicated by a lung abscess requiring antibiotics and thoracic drainage, followed by tapering oral prednisone and initiation of inebilizumab in September 2023. At the latest follow-up, she remained relapse-free with AQP4-IgG seronegativity and her EDSS score of 0.

During hospitalization, the patient received intravenous methylprednisolone at a dose of 80 mg daily for 12 days and intravenous immunoglobulin (IVIG) 22.5 g daily for 5 days, resulting in improvement of dizziness and hiccups. However, she developed a lung abscess presenting with fever, cough, and purulent sputum. The steroid regimen was subsequently tapered, followed by oral prednisone, alongside with anti-infective therapy and thoracic drainage. Following treatment, her symptoms improved significantly. Post-discharge maintenance therapy consisted of oral prednisone, which was gradually tapered and inebilizumab was concomitantly initiated in September 2023. No relapses occurred and AQP4-IgG antibody has been seronegative to date. Her EDSS score was 0 at the last follow-up (Figure 4).

## 3 Literature review and results

**Figure 5:**
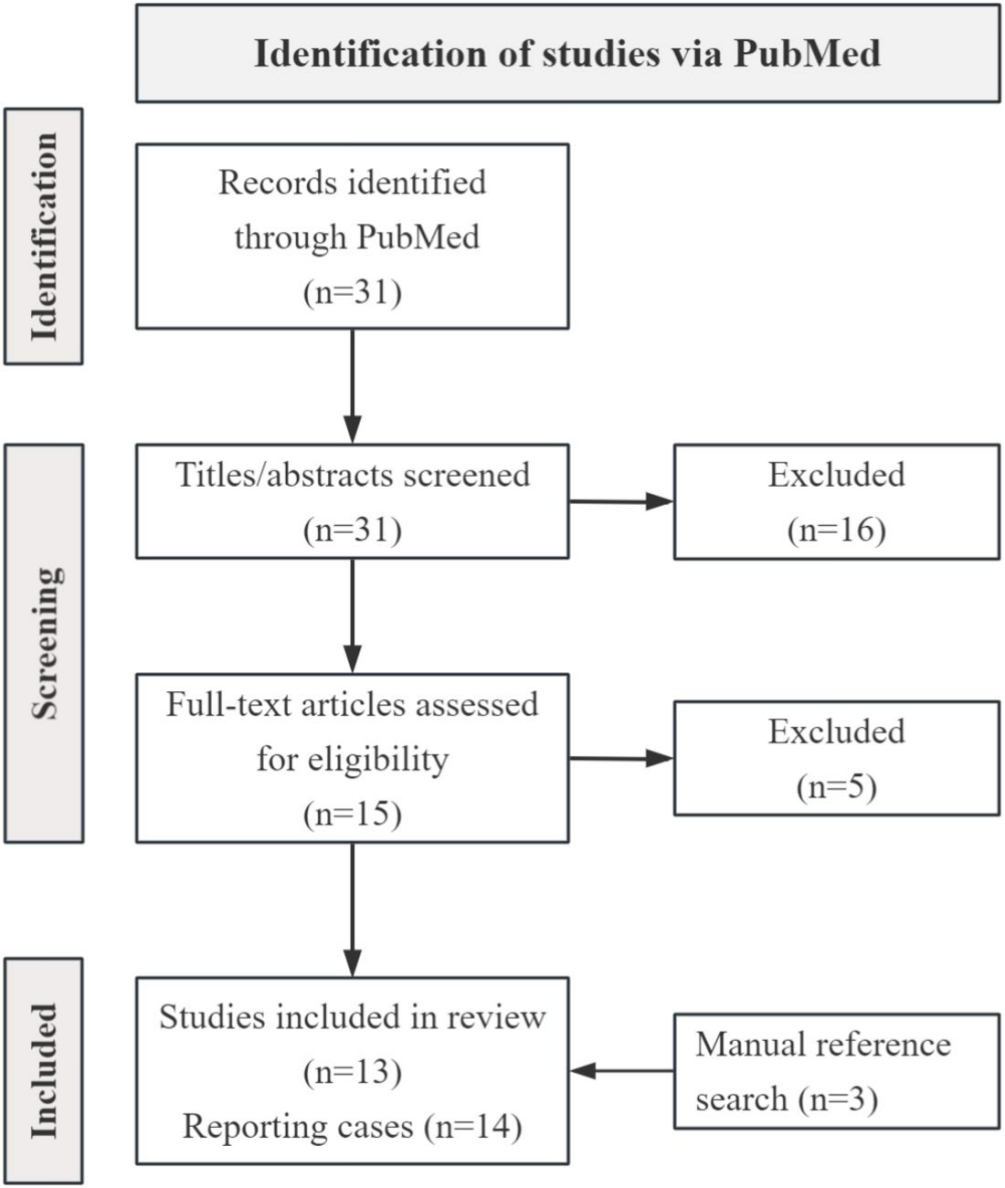
PRISMA flow diagram of the included studies in the review.

We conducted a literature search in PubMed to identify reported cases of NMOSD presenting with syncope. The search strategy combined Medical Subject Headings (MeSH) and free-text terms related to neuromyelitis optica spectrum disorder, area postrema syndrome, hiccups, syncope, loss of consciousness, fainting, sick sinus syndrome, and orthostatic hypotension. Reference lists of relevant articles were also screened. The search yielded 13 studies reporting a total of 14 cases (Figure 5).

Including our two cases, a total of 16 cases were identified in the final cohort (Table 1). The patients ranged in age from 22 to 78 years, with a mean age of 53.94 years. The sex distribution was equal (male-to-female ratio, 1:1). All patients presented with APS. Intractable hiccups (75%) and nausea/vomiting (93.75%) were nearly universal prodromal symptoms preceding syncope attack. Concurrent or subsequent neurological manifestations included limb weakness, brainstem signs, and sensory impairment. Regarding the possible mechanisms of syncope, sinus arrest was documented in eight patients (50%), while orthostatic hypotension was less frequent, occurring in two patients (12.5%). Two patients (12.5%) experienced cardiorespiratory arrest requiring resuscitation. Among the remaining four patients, 24-h Holter monitoring was not performed in three, and in the one patient who was monitored, no arrhythmic events were captured. Although neuro-mediated hypotension could not be entirely excluded in these cases, it was not substantiated by further evidence. Radiologically, MRI findings consistently identified medulla oblongata lesions in all cases. However, as reported by Yin et al. (2025), one patient with severe APS symptoms and recurrent syncope showed no significant lesions on the initial brain MRI upon admission. A subsequent high-resolution scan showed a faint dorsal medullary hyperintensity.

**Table 1:** Patients with AQP4-IgG positive NMOSD presented with syncope.

Reference	Age/Sex	Clinical presentation	APS	Syncope characteristics	Suspected syncope mechanism	AQP4-igg status	MRI lesions	Acute treatment	Maintenance therapy	Outcome
Yin et al. (2025)	70/F	Nausea, hiccups, vomiting	Yes	Recurrent syncope, sinus arrest on ECG	Arrhythmic	Positive	Dorsal medulla oblongata	IVMP, eculizumab	Oral PSL, eculizumab	No syncope attacks
Wang et al. (2024)	34/F	Hiccups, nausea, vomiting	Yes	Recurrent syncope, sinus arrest on ECG	Arrhythmic	Positive	Dorsal medulla oblongata, cervical cord	IVMP, TPM, CNA	Oral PSL	TPM removed, no syncope attacks
Lin et al. (2023)	45/M	Hiccups, vomiting	Yes	Recurrent syncope, sinus arrest on ECG	Arrhythmic	Positive	Dorsal medulla oblongata	IVIG, IVMP, TPM	Rituximab	TPM removed, no syncope attacks
Lin et al. (2023)	26/M	Hiccups, vomiting, dysphagia, diplopia, limb weakness	Yes	Respiratory and cardiac arrest	Arrhythmic	Positive	Dorsal medulla oblongata, pons, mesencephalon	IVMP, PE, IVIG	Oral PSL, azathioprine	No syncope attacks
Hamaguchi et al. (2022)	77/F	Nausea, vomiting	Yes	Recurrent syncope, sinus arrest on ECG	Arrhythmic	Positive	Dorsal medulla oblongata, cervical cord	IVMP, PE, PPM	Oral PSL	No syncope attacks
Tsouris et al. (2020)	42/M	Hiccups	Yes	Recurrent syncope, sinus arrest on ECG	Arrhythmic	Positive	Caudal medulla oblongata	IVMP, pacemaker	Oral PSL	No syncope attacks
Endo et al. (2020)	22/F	Nausea	Yes	Recurrent syncope, sinus arrest on ECG	Arrhythmic	Positive	Dorsal medulla oblongata	IVMP, TPM	Oral PSL	TPM removed, no syncope attacks
Komaki et al. (2020)	77/M	Hiccups, vomiting	Yes	Single Syncope, sinus arrest on ECG	Arrhythmic	Positive	Dorsal medulla oblongata	TPM	Oral PSL	TPM removed, no syncope attacks
Okada et al. (2019)	61/F	Hiccups, nausea, vomiting	Yes	Recurrent syncope, sinus arrest on ECG	Arrhythmic	Positive	Dorsal medulla oblongata	IVMP	Oral PSL	No syncope attacks
Ikumi et al. (2015)	68/F	Hiccups, vomiting	Yes	Recurrent syncope, OH	Autonomic/reflex	Positive	Dorsal and lateral medulla oblongata	IVMP	Oral PSL	No syncope attacks
Sawai et al. (2021)	74/M	Hiccups, nausea, vomiting	Yes	Recurrent syncope, OH	Autonomic /reflex	Positive	Dorsal medulla oblongata	IVMP	Oral PSL	No syncope attacks
Okada et al. (2012)	78/M	Nausea, hiccups, muscle weakness	Yes	Two syncope attacks, OH, respiratory and cardiac arrest	Mixed	Positive	Medulla oblongata, cervical cord	IVMP, IVIG	N/A	No syncope attacks
Zhao et al. (2024)	64/F	Sudden dizziness, nausea, vomiting	Yes	Recurrent syncope	Undetermined	Positive	Dorsal medulla oblongata	PE	N/A	No syncope attacks
Zhou and Luo (2019)	44/M	Hypersomnia, nausea, vomiting	Yes	Recurrent syncope	Undetermined	Positive	Dorsal medulla oblongata, hypothalamus, aqueduct of midbrain	IVMP	Oral PSL	No syncope attacks
Our patient	40/M	Nausea, vomiting, hiccups	Yes	Recurrent syncope	Undetermined	Positive	Medulla oblongata, cervical cord	IVMP	Inebilizumab	No syncope attacks
Our patient	41/F	Sudden dizziness, hiccups, nausea, vomiting	Yes	Two syncope attacks	Undetermined	Positive	Dorsal medulla oblongata	IVMP, IVIG	Inebilizumab	No syncope attacks

TPM, temporary pacemaker implantation; CNA, cardioneuroablation; IVMP, intravenous methylprednisolone pulse; PSL, prednisone; PE, plasma exchange; PPM, permanent pacemaker implantation; IVIG, intravenous immunoglobulin; OH, orthostatic hypotension; N/A, not available.

Acute immunomodulatory therapies were administered to 15 patients (93.75%), including IVMP, IVIG, and plasma exchange (PE), either alone or in combination, leading to substantial symptom improvement in the majority of cases. Overall, the treatment outcomes indicate a high responsiveness to immunotherapy. Cardiac interventions included temporary pacemakers (TPM) in 4 of the 8 sinus arrest cases (50%) and permanent pacemaker (PPM) implantations in 2 cases (25%). Notably, one of the patients who received a TPM also underwent cardioneuroablation (CNA). All TPM recipients were successfully removed without syncope recurrence. Interestingly, two patients showed marked improvement with IVMP monotherapy alone without cardiac intervention. This may indicate that syncope may be driven, at least in part, by inflammatory demyelination in AP and medullary autonomic nuclei and could be reversible once acute inflammation is controlled. During follow-up, all 16 patients remained free of syncope.

## 4 Discussion

NMOSD typically affects the optic nerves, spinal cord, and AP (Weinshenker and Wingerchuk, 2014; Lennon et al., 2004). Beyond its classic core syndromes (Rosales and Kister, 2016), autonomic dysfunction has increasingly been recognized as an important clinical feature (Andabaka et al., 2023). Reported manifestations are diverse and extend beyond cardiovascular symptoms, including orthostatic intolerance, pupillomotor abnormalities, gastrointestinal dysmotility, bladder and bowel dysfunction, and sudomotor or vasomotor symptoms (Yang et al., 2023). Among these, cardiovascular autonomic dysfunction (CAD) is a critical subset of autonomic dysfunction, characterized by impaired blood pressure regulation, blunted heart rate variability, and orthostatic intolerance (Crnošija et al., 2020). While CAD is frequently observed in multiple sclerosis (MS), emerging evidence suggests that CAD in NMOSD warrants comparable clinical attention. According to Crnošija et al. (2020), compared with patients with MS, CAD in NMOSD is more severe when present and often manifests as orthostatic hypotension (OH) and parasympathetic dysfunction. The syncope cohort predominantly exhibited severe, parasympathetic-mediated arrhythmias. This distinctive pattern is plausibly attributable to the predilection of NMOSD for the thoracic spinal cord and brainstem, which are the most critical central regions for autonomic regulation. Although MS can involve the medulla oblongata, such involvement is typically partial, asymmetric, and occurs with gradual progression, resulting in a lower proportion of severe CAD (Adamec et al., 2018).

In recent years, with the increasing availability of AQP4-IgG testing, syncope has been increasingly reported as a clinical manifestation of NMOSD. Notably, Yin et al. (2025) described an AQP4-IgG-positive patient without detectable medullary lesions on initial brain MRI, highlighting the critical role of serological testing. In NMOSD, syncope often occurs in the context of APS and medullary involvement, suggesting that APS and syncope may share a common anatomical basis. Traditionally, syncope is considered as a manifestation of primary cardiac disorders, but it can arise from multiple etiologies, including neurogenic, and reflex-mediated mechanisms (da Silva, 2014). In our review of 16 cases, 10 exhibited arrhythmic mechanisms (sinus arrest in eight, cardiopulmonary arrest in two), and two had autonomic/reflex (OH). Consequently, CAD may play a central role in the pathogenesis of syncope with NMOSD.

**Figure 6:**
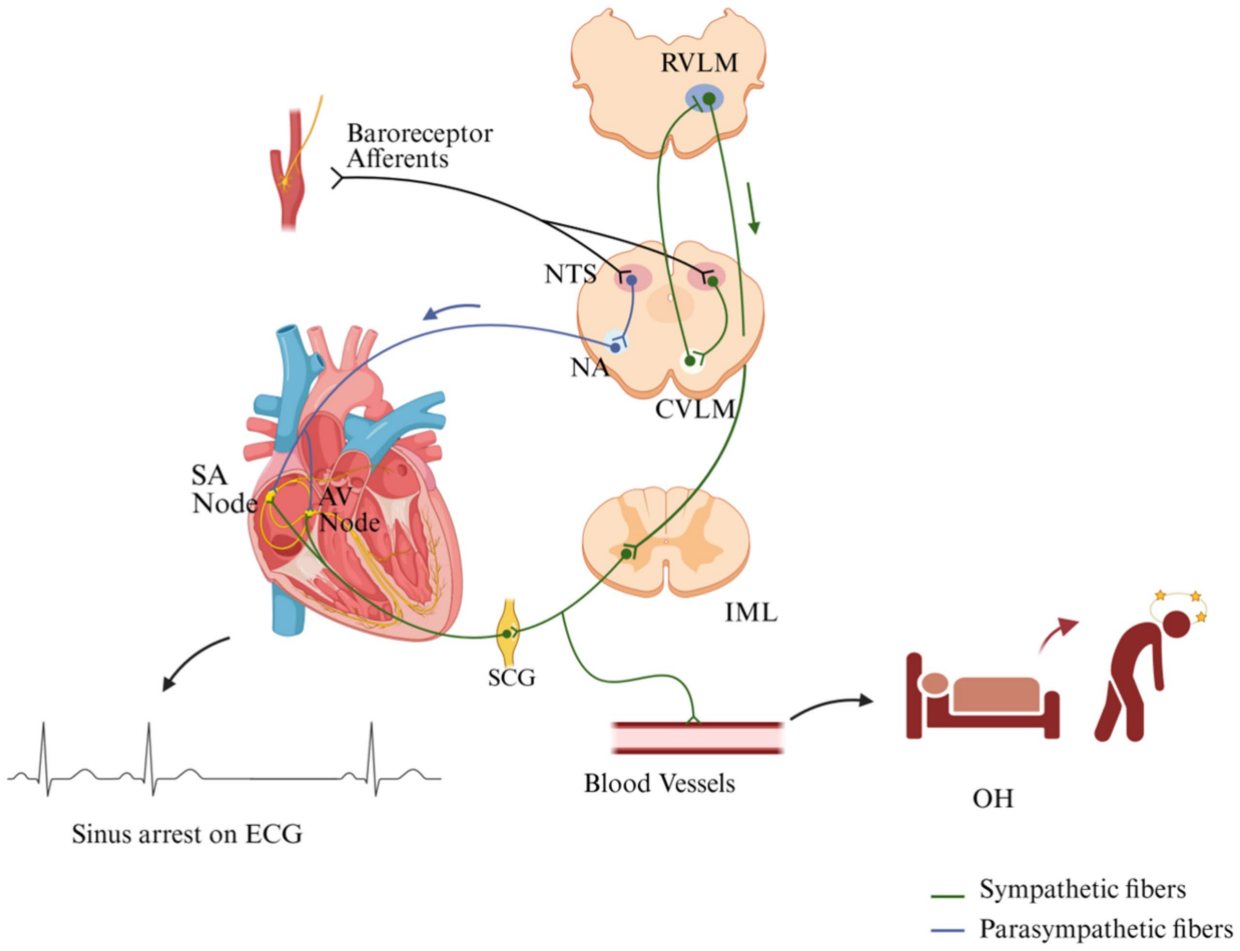
Cardiac autonomic regulation: sympathetic and parasympathetic pathways. The nucleus tractus solitarius (NTS) serves as the primary relay for baroreceptor afferents, integrating peripheral cardiovascular signals. These signals are transmitted to the rostral ventrolateral medulla (RVLM) and nucleus ambiguus (NA). The RVLM activates sympathetic fibers via the intermediolateral column (IML) and sympathetic chain ganglia (SCG), increasing SA node firing rate, AV node conduction velocity, and vascular tone. Conversely, the NA inhibits SA node activity through vagal parasympathetic fibers. The caudal ventrolateral medulla (CVLM) modulates RVLM activity via inhibitory input. Disruption of this pathway may result in sinus arrest and orthostatic hypotension (OH). (Created with BioRender.com).

Prior case series indicate that syncope in NMOSD may stem from structural disruption of medullary autonomic nuclei, specifically the nucleus tractus solitarius (NTS) and rostral ventrolateral medulla (RVLM). The NTS, situated in the dorsal medulla, acts as the primary relay station for baroreceptor afferents, integrating cardiovascular signals from peripheral arterial sensors (Cutsforth-Gregory and Benarroch, 2017). These signals are then transmitted to the RVLM and the nucleus ambiguus (NA), which collectively regulate heart rate and vascular tone (Palma and Benarroch, 2014). The RVLM is a key integrative center for sympathetic outflow. Located in the medulla oblongata, it receives input from cardiovascular regulatory pathways and projects to the intermediolateral column (IML) of the thoracic spinal cord. From there, sympathetic preganglionic neurons relay signals through the sympathetic chain ganglia (SCG) to peripheral effectors. The NA relays parasympathetic output through vagal fibers to the sinoatrial (SA) node, while the RVLM activates sympathetic cardiac nerves via the sympathetic trunk to increase SA node firing rate and atrioventricular (AV) node conduction velocity, thereby elevating heart rate and contractile force. Additionally, the caudal ventrolateral medulla (CVLM) modulates RVLM activity, providing inhibitory control over sympathetic tone (Mandel and Schreihofer, 2008) (Figure 6). In NMOSD, lesions involving the medullary tegmentum may damage this circuitry, leading to baroreflex failure. Accordingly, the presence of medullary lesions should prompt careful monitoring for syncope and other manifestations of CAD.

Furthermore, AP plays a vital role in controlling the vomiting reflex and other autonomic functions, appetite regulation, and somatic growth (Sarnat et al., 2019). As a circumventricular organ lacking a complete blood–brain barrier, the AP is highly vulnerable to circulating AQP4-IgG (Raj et al., 2023). Antibody binding triggers plasma interleukin-6 (IL-6) release and complement-mediated astrocyte injury, leading to inflammatory edema and subsequent dysfunction of adjacent nuclei, including the NTS (Bruscolini et al., 2018). Given that the NTS also integrates inputs driving the vomiting reflex, we propose that syncope in NMOSD may be viewed as an extension of the same pathophysiological process that underlies APS, rather than a separate comorbidity.

Our analysis also provides several clinically relevant insights for risk stratification and monitoring. Although the total number of reported cases is small, most patients were middle-aged to elderly, without clear sex predominance. Advanced age may be a non-negligible risk factor for clinically significant autonomic complications. Continuous ECG and respiratory monitoring may be warranted for patients with medullary lesions, particularly in those with active infection, hemodynamic instability, or during IVMP pulse therapy.

Immunosuppressive therapy is crucial for controlling NMOSD progression, as NMOSD-related cardiac dysfunction may be reversible once acute inflammation is controlled. Prior studies have reported that some patients with sinus arrest were successfully managed with immunosuppressive therapy alone, without pacemaker implantation (Okada et al., 2019). In addition, for the minority of patients presenting with life-threatening cardiorespiratory arrest, a more aggressive management strategy appears necessary. The reviewed cases suggest that successful outcomes rely on the rapid initiation of high-dose IVMP combined with IVIG or PE. In cases of severe bradycardia or sinus arrest, temporary pacing may serve as a bridge to recovery until immunomodulatory therapy takes effect (Podoleanu and Deharo, 2019). For long-term disease control, maintenance oral corticosteroids remain standard in many settings, while emerging biological agents are expanding therapeutic option. Both patients in our series received inebilizumab, and achieved sustained clinical stability without relapse, with seroconversion to AQP4-IgG negativity.

This study has several limitations inherent to its design. First, as a synthesis based primarily on published case reports and small case series, our findings are susceptible to publication bias, which may overestimate the prevalence of syncope in the general NMOSD population. Second, in our two patients, a systematic assessment of autonomic function was not performed, as both presented with stable vital signs and had no further episodes of syncope at the time of our evaluation. Notably, heterogeneity in the clinical evaluation and reporting details across the included cases limits direct comparisons and pooled analyses. Moreover, the observational nature of case-based evidence prevents definitive conclusions regarding causality. Although medullary lesions are frequently observed in patients with autonomic symptoms, the precise pathophysiological mechanisms remain inferential.

## 5 Conclusion

In summary, syncope may represent an underrecognized manifestation of APS in patients with AQP4-IgG-positive NMOSD, particularly when medullary lesions are present. The presence of APS symptoms should prompt heightened clinical vigilance for syncope and even cardiorespiratory arrest. Furthermore, in patients presenting with unexplained syncope accompanied by persistent nausea, vomiting, or hiccups, NMOSD should be considered as an important diagnosis.

## Data Availability

The original contributions presented in the study are included in the article/supplementary material, further inquiries can be directed to the corresponding author.
